# Quick, Safe, Effective: Ultrasound-Guided Percutaneous Liver Biopsy in the Pediatric Patient

**DOI:** 10.1007/s00270-023-03631-7

**Published:** 2023-12-21

**Authors:** Russell M. Salamo, Joseph Miller

**Affiliations:** 1 Department of Radiology, LAC+USC Medical Center, 2051 Marengo St., Los Angeles, CA 90033 USA; 2 Department of Radiology, Children’s Hospital Los Angeles, USC, Los Angeles, CA USA

**Keywords:** Pediatrics, Hepatobilliary

## Abstract

**Background:**

Percutaneous liver biopsy has proven to be a valuable tool in the workup of pediatric acute liver failure and the management of post-transplant rejection. However, consensus regarding pre-procedure laboratory values and post-procedure monitoring is lacking.

**Objective:**

To characterize the incidence of complications, procedural time, and specimen adequacy for percutaneous liver biopsy in the pediatric patient.

**Methods:**

Retrospective review of percutaneous liver biopsies at a single institution was performed for a 5-year span. Procedural notes and anesthesia records were sampled for patient weight and procedural factors across a continuous 6-month period, as well as for the subgroup of patients under 24 months of age. A representative continuous subset of pathology reports comprising 376 patients were reviewed for estimation of specimen adequacy.

**Results:**

Eight hundred and sixty-seven ultrasound-guided percutaneous liver biopsies were performed in a 5-year period, 450 of which were in the post-transplant setting with about a 3:1 ratio of split: whole liver transplant. Patient ages ranged from 1 month to 21 years old, with weight ranging from 2.7 to 125 kg. Of the 376 pathology reports available, none were found to be inadequate for evaluation. Two major complications occurred, both of which were biliary leaks in the setting split-liver transplant. There were no incidences of post-procedure hemorrhage. Of the sample reviewed, mean “skin-to-skin” procedure time was under 8.5 min (median of 7 min). Solely among transplant patients, biopsies for split livers averaged 9.2 min, biopsies for whole livers averaged 6.2 min (two-tailed independent *t* test, *p* = 0.0426).

**Conclusion:**

Ultrasound guided percutaneous liver biopsy is fast, useful, and safe in pediatric patients on an outpatient basis with same day discharge.

**Level of Evidence IV:**

This journal requires that authors assign a level of evidence to each article. For a full description of these Evidence-Based Medicine ratings, please refer to the Table of Contents or the online Instructions to Authors www.springer.com/00266.

## Introduction

Despite the risks inherent to any solid organ biopsy, there is often no substitute for histopathology in the management of pediatric liver disease [1]. In the pediatric population, image-guided percutaneous liver biopsy has demonstrated advantages to other approaches in a number of clinical situations, particularly in small patients where transcatheter endovascular biopsy may be technically challenging [2].

We present a pediatric cohort of ultrasound-guided percutaneous liver biopsies performed by interventional radiologist with the purpose of characterizing the incidence of complications, technical aspects, and suitability for same day discharge.

## Materials and Methods

### Data Collection

Institutional review board approval was obtained for this retrospective review. A continuous 5-year period was selected extending from July 1, 2017 to June 30, 2022. For each procedure, the patient’s age and transplant status were recorded. All procedural complications were followed prospectively at the time of procedure, and again by retrospective chart review. No routine post-biopsy follow-up was scheduled or performed. A representative continuous subset of pathology reports comprising 376 patients were reviewed for estimation of specimen adequacy. Adequacy was defined according to the explicit text of the pathologist report itself. No reports were seen to use the words “inadequate” or “nondiagnostic” (or similar), nor were any reports without a pathologic diagnosis.

Procedural notes were sampled for all biopsies performed across a continuous 6-month period. Patient weight at time of biopsy, total procedure time (“skin-to-skin” for the operating physician), size of coaxial biopsy system utilized, number of core biopsies obtained, and approach (right intercostal/subcostal or left subcostal) were recorded. Indications for biopsy and the final diagnosis was also recorded. A similar analysis was performed for a subgroup of all patients aged 24 months and younger.

### Inclusion Criteria

All liver biopsies performed during the study period were evaluated. Patients were evaluated for percutaneous biopsy via the society of interventional radiology (SIR) periprocedural guidelines for biopsy of solid organ or deep non-organ. For all patients, an international normalized ratio (INR) was less than or equal to 1.8 and platelet count was greater than or equal to 50,000. Transcatheter biopsies were reserved for patients with uncorrectable coagulopathy only.

Peri-procedural antibiotics were not routinely provided, and however, patients with known or suspected cholangitis were continued on their antibiotic therapies prior to and after biopsy.

### Procedural Technique

Biopsy procedures were performed with a Bard Mission Disposable Core Biopsy Instrument (Bard, Tempe, Arizona) in either 18 G × 10 cm (17 G × 5.1 cm outer coaxial cannula needle) or 20 G × 10 cm (19 G × 5.1 cm outer coaxial cannula needle) size. This is a semi-automatic side-coring coaxial biopsy instrument.

As is standard practice at our free-standing tertiary care pediatric institution, all patients received general anesthesia with medication administration and airway management at the discretion of the anesthesiologist. Whenever possible, a laryngeal mask airway (LMA) was placed following induction of anesthesia. In those instances where the patients clinical conditioned precluded LMA, intubation was performed. Conscious sedation was not utilized.

A right-sided approach to the liver was taken whenever possible, with preference given to subcostal over intercostal approaches. In the setting of a split-liver transplant, a left-sided subcostal approach was typically taken.

Using real-time ultrasound guidance, the subcutaneous tissues were anesthetized with 0.25% bupivacaine to a maximum of 1 mL per kilogram of patient body weight. Without use of a dermatotomy, the outer coaxial cannula needle was passed under continuous ultrasound guidance into the liver parenchyma. The needle was advanced to ensure the cannula tip was positioned at least 5 mm deep to the liver capsule. The semi-automatic biopsy instrument was then inserted into the cannula with continuous ultrasound visualization as the side-cutting biopsy tray was deployed and fired. The coaxial system allows for multiple passes of the side-coring needle through a single capsular defect. Standard practice was to obtain 2 cores at 2 cm length when possible, however more or less may be obtained in any case depending on operator preference or clinical need. Neither post-biopsy local compression or banding was performed.

### Recovery

Patients were routinely monitored for 4 h in the post-anesthesia care unit (PACU), with supine bedrest and kept nothing by mouth (NPO) for the first two. Vitals were checked every 15 min for the first hour, and then every 30 min for the following 3 h thereafter. Patients too young to reliably remain flat on their own once awake were often either given additional sedative medications (dexmedetomidine or propofol) or held in the arms of their parent or a PACU nurse until the first 2 h had elapsed. Overnight admission and serial laboratory checks (i.e., hemoglobin) were not routinely practiced.

## Results

Over the course of the 5-year period studied, 867 ultrasound-guided percutaneous liver biopsies were performed. Patient ages ranged from 1 month to 21 years old, with weight ranging from 2.7 to 125 kg. A total of three interventional radiologists performed the procedures, with approximately 60% performed by a single operator. Patient demographics are outlined in Table 1. 450 of the biopsies were in the post-transplant setting with about a 3:1 ratio of split: whole liver transplant.

**Table 1:** Patient Demographics; (*N *= 867)

Median age (years)	12 (0.09–21)
Gender	
Male (%)	55 (476)
Female (%)	45 (391)
Median weight (kg)	32.3 (2.7–125.5)
Clinical status	
Inpatient	44.8 (389)
Outpatient	55.2 (478)

At the time of review, a continuous representative sample of 376 pathology reports were readily available for evaluation (Table 2). The 376 samples reported represent the extant reports produced as part of an institutional quality improvement process and were utilized for their ease of analysis. None of the provided tissue samples were found to be inadequate for evaluation by pathology.

**Table 2:** Indications for biopsy

Transplant evaluation (%)	55.3
Obesity/NASH evaluation (%)	29.0
Autoimmune hepatitis (%)	5.3
Hepatitis NOS (%)	4.0
Neonatal hyperbilirubinemia (%)	2.7
Mass lesion (%)	2.1
Acute liver failure (%)	1.6

Two adverse events occurred during the studied period. Both patients had undergone split-liver transplantation and underwent percutaneous biopsy for suspicion of severe acute rejection. Neither patient had evidence of complication during the biopsy or in the immediate post-operative period. Both patients developed biliary leak approximately 2 weeks after biopsy in the setting of prolonged high-dose steroids, despite a lack of either prospective or retrospective evidence of biliary duct traversal by the biopsy needle. No bleeding complications were identified in either the acute or delayed post-procedural period. None of the patients required transfusion of blood products or unexpected admission following biopsy.

Of the sample reviewed, mean “skin-to-skin” procedure time was under 8.5 min (median of 7 min). Solely among transplant patients, biopsies for split livers averaged 9.2 min, biopsies for whole livers averaged 6.2 min (two-tailed independent *t*-test, *p* = 0.0426). Induction of anesthesia and the reported 4 h post-procedural monitoring were not considered for the calculated procedure time.

## Discussion

Gastroenterologists and surgeons continue to be the specialties primarily providing liver biopsy services in the pediatric population [3, 4]. This likely owes in part to the limited number of dedicated pediatric interventionalists, with recent report demonstrating only 125 across the USA, with significant concentration in a handful of centers [5]. There is currently no literature comparing relative complication rates between the techniques employed between these specialties, however we believe that our complete lack of primary procedural complications among over 850 patients ranging from 2.7 to 125 kg across all-comers within a 5-year period speaks highly to the value of the interventional radiologist in this patient population. This suggests a strong need for additional board-certified interventional radiologists in the setting of pediatric care.

Ultrasound-guided percutaneous liver biopsy can lead to a variety of complications including bleeding, sepsis, bile leak, hemobilia, arterioportal fistula, pneumothorax, hemothorax, or injury to other adjacent organs [6]. The reported incidence of complications is felt to be between 0.20 and 2.90% [7]. We report no deaths or hemorrhages, and two delayed-onset complications (both bile leaks that developed 2-weeks post biopsy in the setting of prolonged high dose steroid administration), a rate of 0.20%.

The reported incidence of bleeding after percutaneous liver biopsy is 0.26–2.58% [6, 7]. We report no incidence of post-biopsy hemorrhage and at no point was GelFoam tract injection, manual compression, hemoglobin trending, or overnight admission for observation performed. The SIR characterizes percutaneous liver biopsy as a high bleeding risk procedure and has recommended that a platelet count should be 50,000 or higher, and the INR should be 1.8 or lower prior to biopsy [8]. The European society for pediatric gastroenterology hepatology and nutrition (ESPGHAN) Hepatology Committee overall suggests that an INR value above 1.5 should warrant factor transfusion or biopsy via a different approach (transvenous or laparoscopic). A consensus on platelet threshold was not reached, but 60,000 was suggested [9]. However, there is a lack of correlation between these parameters and the reported bleeding risk. A comparison study between transcatheter endovascular liver biopsy and percutaneous liver biopsy with tract embolization found no statistically significant difference in complications [10]. Moreover, no significant difference has been found between coaxial and non-coaxial approach or between the coaxial method with or without injection of absorbable gelatin pledgets [11].

The SIR characterizes transcatheter endovascular liver biopsy in adults as a low-risk bleeding procedure [8]. However, there are several potential pitfalls associated with this technique in pediatric recipients. Additionally, there is an inherent technical challenge due to the frequent size mismatch between the equipment and the patient. In pediatric liver transplant recipients, the small volume of the engrafted organ and altered angle between the hepatic vein and inferior vena cava can further limit the endovascular approach. This is compounded further with split-liver transplants where, unlike whole liver transplant recipients, the number of hepatic veins is limited with a left lobe or lateral segment [12]. If portal or hepatic vein pressure measurements are requested, then transcatheter endovascular approach to liver biopsy has typically been preferred over percutaneous. However, it is accepted that pressure measurements can be performed through internal jugular or femoral access with a small 4- or 5-French sheath and then liver biopsy can subsequently be performed percutaneously in small patients.

The incidence of sepsis after percutaneous liver biopsy in non-immunocompromised hosts has been reported to be 0.01% [13]. Pediatric patients who are post-liver transplant are at an increased risk of developing sepsis after liver biopsy due to immunocompromised state, the likely presence of a hepaticojejunostomy as part of a split-liver transplant, and the relatively high incidence of chronic biliary complications in the setting of biliary reflux and cholestasis. The reported incidence of sepsis following liver biopsy in the immunocompromised, post-transplant patient is 0.32–1.84% [6, 7]. Prophylactic antibiotics were not administered, as was done in other reported studies (although antibiotic courses initiated for suspected or known cholangitis were continued peri-operatively), and we report no incidences of post-biopsy sepsis.

It has been suggested that practice guidelines, with specific attention to overnight hospital observation, for the monitoring of pediatric patients post liver biopsy deserve re-evaluation [14]. However, the most recent and largest reported global cohort of pediatric patients routinely practiced overnight hospital admission following biopsy. At our institution, patients are routinely discharged (or returned to their inpatient room without additional observation) after 4 h of post-procedure monitoring, regardless of their weight or age.

Published adult liver biopsy literature suggests a diagnostic yield of 98% when performed by an interventional radiologist. However, pediatric studies have described diagnostic accuracy of only 86% [9]. While this discrepancy is likely due to a number of factors, the aforementioned fact that most pediatric liver biopsies reported in the literature are not performed by IR (and therefore not necessarily with an image-guided technique) could play a major role. Across our reviewed pathology reports, none were found to be inadequate for evaluation across multiple procedural indications.

The main limitations of the current study are its retrospective nature. In addition, pathology reports were not evaluated for all 867 biopsies performed. However, this representative cross-sectional period of the study was chosen to generate descriptive statistics about the procedure itself (procedural time, etc.). As such, 6 months should be considered extensive enough to average out any potential outliers.

In conclusion, ultrasound guided percutaneous liver biopsy can be done safely in the outpatient setting with same day discharge. The procedure is fast and useful for a variety of indications. The bleeding risk is likely lower than what is currently promoted and further data for a uniform consensus regarding laboratory values are warranted.
